# The American Hospital Association

**Published:** 1910-11-19

**Authors:** 


### SPECIAL INSTITUTIONAL ARTICLES.

### THE AMERICAN HOSPITAL ASSOCIATION.

### TWELFTH ANNUAL CONFERENCE.

This Conference, which was held at St. Louis, Mo., on September 20, 21, and 22 last, was well attended, and the papers read were adequate to the occasion both in quantity and quality.

Dr. C. Irving Fisher, Superintendent of the Presbyterian Hospital, New York City, contributed a paper on "The Need of an Intermediate Single-room Service in Hospitals," which we hope to publish in full in The Hospital. He pointed out that in American hospitals the needs of the poor and rich are well provided for, as in all hospitals on this side of the Atlantic private-room service is to be obtained; but the "middle class," the skilled artisans and mechanics, small merchants, literary people, artists, teachers, ministers, and the great army of salaried people are not catered for. True these can go into a public ward and pay, but the majority do not like to do this. The private room is beyond the limit of their means, and thus they must perforce shoulder the burden of sickness in their own homes, usually under unfavourable sanitary conditions. Therefore Dr. Fisher advocates the establishment in city hospitals of plain single rooms, or of very small wards, with a service a little better than the ward service, at a moderate fee. There might also be added a moderate fixed fee for the attendance of physician or surgeon, corresponding to the regular charge of the medical men whom this class of patient employs outside. Dr. Fisher drew attention to the fact that one of the elements of the fame and success of the well-known Mayo Brothers, of Rochester, Minn., has been the care taken to investigate very strictly patients' circumstances and to establish a scale of fees fair to all concerned. Such methods should be possible in all hospitals.

Mr. Bailey B. Burritt, Assistant Secretary, State Charities Aid Association, New York City, read a paper on "Co-operation *versus* Individualism in the Care of the Sick." This able paper was a plea for the municipalisation of hospitals.

Dr. S. S. Goldwater, Superintendent, Mount Sinai Hospital, N.Y., read a paper on "Hospital Planning in Crowded Centres of Population," and propounded plans whereby space might be economised without detriment to the health of the patients.

Dr. Henry M. Hurd, Superintendent of the Johns Hopkins Hospital, Baltimore, read a paper on "The Proper Relationship of the Superintendent to the Trustees of a Hospital." Dr. Hurd argued that as the superintendent of a hospital is no longer selected for the position because he has been a failure in some other branch of business, he should be a thoroughly good business man and should be given large powers. He regrets that as yet there is no systematic training of men and women to fill such positions, but he thinks that the Hospital Association owes it to the country to arrange for courses in hospital administration to fit men and women for efficient service.

Dr. R. O. Beard, Director of the Department of Physiology and Pharmacology and Secretary of the Hospital of the University of Minnesota, read a paper on "The Education of the Nurse in America." This paper was an eloquent plea for the more systematic training of women nurses than obtains in the United States at the present time.

Dr. H. B. Howard, in addition to the presidential address, contributed an interesting paper on the State Farm, Mass., of which institution he is in control. The "State Farm" is an institution composed of three classes of inmates. The first class are misdemeanants committed by the municipal, police, and district courts of Massachusetts, the principal offenders being drunkards, vagrants, and tramps; these number about 1,500. The second class are criminal insane men, about 700. Third class, State paupers, about 300; or a total of 2,500 or thereabouts. There is a farm of 1,200 acres or so. Dr. Howard gave an exhaustive description of how he evolved order out of chaos and brought a badly managed institution, of which the buildings were of wood, into a state of high efficiency, with ample fireproof buildings, in the space of twenty-seven years and at a very moderate cost.

Mr. Clarence W. Williams, engineer, Boston, Mass., read a paper on "Modern Practice in Hospital Heating and Ventilation." Mr. Williams said that Benjamin Franklin was undoubtedly the originator of what has been commonly recognised as American practice of heating and ventilation. Between the years 1740 and 1745 he invented what he termed the Pennsylvania fireplace, an apparatus which took cold fresh air from outside the building, and after tempering it or warming it in passages heated by the escaping gases of the fire finally discharged it into the room. The principle of the Pennsylvania fireplace is still adhered to by most heating engineers throughout the country. As for ventilation, Mr. Williams believes in natural ventilation as far as is possible. Mr. Williams, in concluding his paper, offered the suggestion that there should be included in the course of instructions for nurse training at least one lecture during the early autumn months upon heating and ventilation of hospital buildings and methods of control, so that when these young women have become qualified nurses and responsible for such problems they will not be wholly ignorant of the important subjects of heating and ventilation.

The report was read of a committee appointed to consider the education and training of nurse assistants for the care of people of moderate means in their homes and the nursing of patients suffering from chronic diseases. The committee have thoroughly looked into and discussed the matter, and have concluded that nursing by endowment offers the best solution. The source and general plan of endowment must be determined to a large extent by the local conditions of the community adopting the system. It was thought that possibly some form of local insurance might be devised to assist in paying the cost. Fraternal and benevolent orders, churches, and other organisations doing charitable work would undoubtedly lend their support could they be made to realise that the money invested would be more wisely expended by an especially equipped and organised system than by individual agencies. The patients, of course, should pay such portion of the actual expense incurred as they are able. In those cases in which it is practicable the balance should be paid to suit their convenience.

The question of providing proper treatment and efficient nursing for people with moderate means is a burning one in America, and it is even more insistent in Great Britain. Therefore the plan for supplying private rooms in hospitals at a low cost, and of the endowment of visiting nurses, if put to the test in America, will doubtless be watched with the keenest interest in Great Britain.

It is satisfactory to note that, judging from a perusal of the report, the American Hospital Association is in a flourishing condition.

